# A Case of West Nile Encephalitis: Neuroimaging Findings and Clinico-Radiological Mismatch

**DOI:** 10.7759/cureus.49727

**Published:** 2023-11-30

**Authors:** John Paul Mikhaiel, Amanda Mckenzie, Lea Saab, Adeel S Zubair

**Affiliations:** 1 Neurology, Yale School of Medicine, New Haven, USA

**Keywords:** west nile virus, mri, leukoencephalopathy, encephalitis, wnv

## Abstract

West Nile Virus, an arthropod-borne RNA virus, may result in severe neurological disease. West Nile neuroinvasive disease is characterized by meningitis, encephalitis, and possible acute flaccid paralysis. Classically, signal intensity abnormalities on T2-weighted magnetic resonance images are associated with poor outcomes. Herein, we present a case of previous West Nile encephalitis with diffuse leukoencephalopathy on imaging that demonstrates a favorable clinical outcome with limited neurologic sequelae. A 53-year-old male presented to the hospital with one month of headaches, dizziness, generalized weakness, and a seizure. His initial neurologic exam was notable for wide-based gait and imbalance. Magnetic resonance imaging (MRI) of the brain demonstrated diffuse bilateral white matter signal hyperintensities without contrast enhancement, suggestive of leukoencephalopathy. His lumbar puncture revealed lymphocytic pleocytosis and infectious studies demonstrated positive West Nile Virus immunoglobulin G (IgG) in the cerebrospinal fluid (CSF) and serum with negative immunoglobulin M (IgM) in both CSF and serum, suggestive of previous infection. A diagnosis of sequelae of West Nile neuroinvasive disease was made. He was started on anti-seizure medications without further seizures. At his subsequent nine-month follow-up visit, he remained asymptomatic without weakness, headaches, or confusion. Repeat MRI demonstrated interval improvement of white matter signal change. This case report highlights that West Nile neuroinvasive disease may present with profound white matter changes on MRI with limited clinical symptoms and long-term neurologic sequelae. Further research is needed to identify imaging correlation with symptom severity in this disease.

## Introduction

West Nile Virus (WNV) is a single-stranded enveloped RNA virus and a member of the Flaviviridae family. It is an arthropod-borne virus of which birds are the natural hosts. Humans are incidental hosts and become infected through a mosquito bite, typically of the genus Culex [1]. It was first isolated in 1937 from a patient in Uganda and was among the first arthropod-borne viruses to be detected [2]. It emerged as a public health concern in North America in the late summer of 1999 during an outbreak of human encephalitis and the simultaneous deaths of exotic birds. It is not known precisely where the virus originated from, although a high degree of similarity has been established between US WNV and WNV isolated in Israel a year prior to the outbreak [3].

Most infections remain asymptomatic, however, and 20% of infected patients develop a mild febrile illness known as West Nile fever [4]. It is estimated that less than 1% of infections result in severe neurological disease [5]. West Nile neuroinvasive disease (WNND) is characterized by meningitis and encephalitis, possibly accompanied by acute flaccid paralysis. Encephalitis is more frequently seen in older populations, whereas meningitis occurs more commonly in children. The clinical manifestations of meningitis are like those of other viral meningitides. Encephalitis additionally requires the presence of sustained altered mental status (>24 hours), seizures, or other focal neurologic findings [6]. The fatality rate among patients who meet the clinical criteria for WNND is about 10% [7].

To date, heterogenous findings on magnetic resonance imaging (MRI) of the brain have been described in WNND. In a case series, five of 16 patients demonstrated enhancement of leptomeninges or periventricular regions [7] while other reports showed hyperintensity in the gray matter, including bilateral thalami, caudate nuclei, and lentiform nuclei on fluid-attenuated inversion recovery (FLAIR) or T2-weighted images (T2WI) [8]. Studies have not consistently demonstrated predilection of the virus for gray or white matter, but rather, involvement of both, including cortical and deep gray matter, as well as cerebral and cerebellar white matter [9]. Herein, we present a case of post-infectious West Nile encephalitis with striking MRI brain findings consistent with previous reports of diffuse gray and white matter involvement. 

## Case presentation

**Figure 1:**
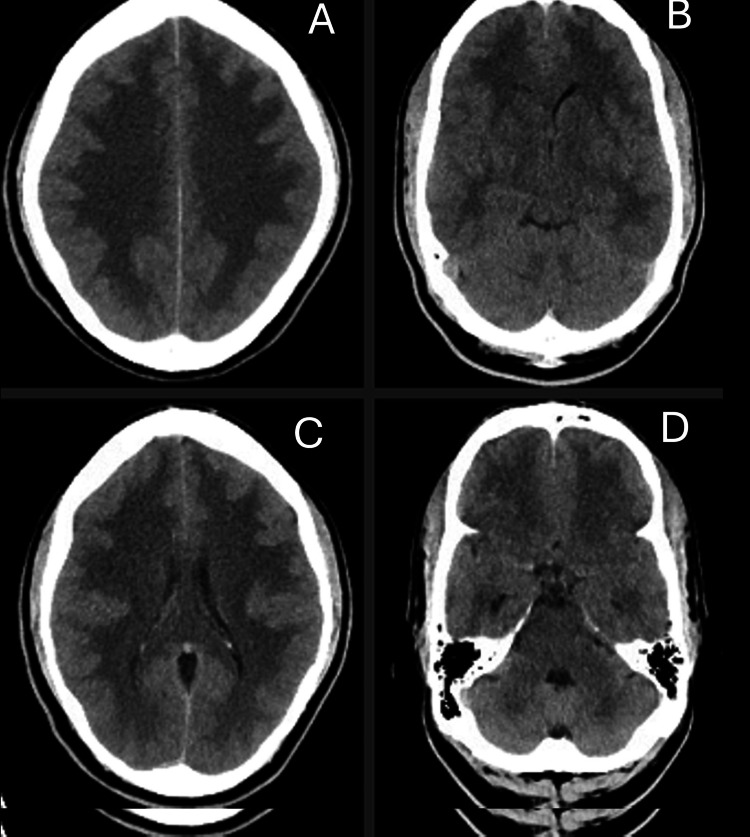
Initial CT scan images of the head Initial CT scan of the head (at the time of presentation) demonstrating diffuse white matter hypoattenuation predominantly involving bilateral frontoparietal (A) and temporal lobes (B) and partial involvement of the occipital lobes (C) and brain stem and cerebellum (D).

**Figure 2:**
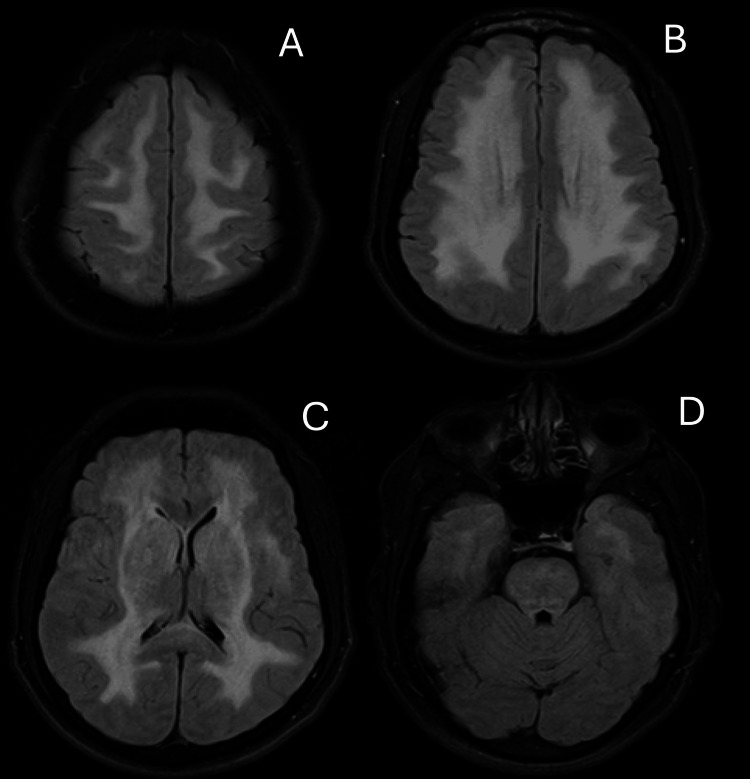
Initial MR imaging of the brain Initial MRI FLAIR sequences (at the time of presentation) demonstrating diffuse symmetric white matter signal abnormality predominantly involving the frontal and parietal cerebral white matter with sparing of the subcortical U fibers (A, B). A bilateral thalamic signal abnormality is present as well as extensive signal abnormality symmetrically within the posterior limbs of the internal capsules and within the external capsules bilaterally (C). There is patchy signal abnormality throughout the pons and within the dentate nuclei (D).

A 53-year-old male with no significant past medical history presented to the hospital with one month of headaches, dizziness, and generalized weakness. His initial neurologic examination was notable for wide-based gait and imbalance but was otherwise within normal limits. On the day of his presentation, he had an episode of near syncope for which he underwent computed tomography (of the head) that demonstrated diffuse hypoattenuation of the subcortical white matter of the frontal, parietal, and temporal lobes as well as the thalami, midbrain, and pons regions (Figure 1). MRI of the brain was consistent with leukoencephalopathy (Figure 2). Peripheral blood work did not reveal evidence of inflammation without leukocytosis or elevated acute phase reactants. Lumbar puncture (LP) revealed one red blood cell (RBC), seven nucleated cells with 95% lymphocytes, and normal protein and glucose, consistent with lymphocytic pleocytosis with negative infectious studies, including treponema pallidum, human immunodeficiency virus, Epstein-Barr virus, enteroviruses, JC virus, and herpesviruses. West Nile Virus panel was sent but pending on discharge. The patient was feeling well without residual headache, weakness, and dizziness, and so was discharged with a plan for outpatient follow-up for the remaining CSF studies. No medications were started at this time.

**Figure 3:**
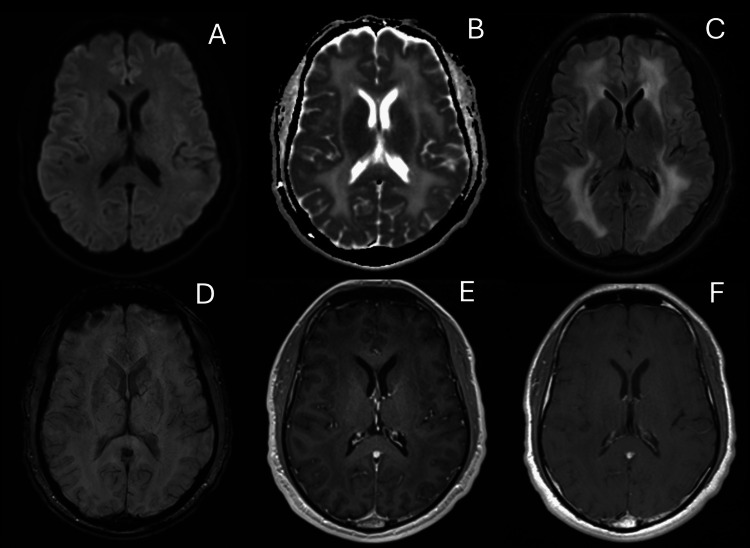
Repeat MRI of the brain Repeat brain MRI, one week after initial presentation. (A, B) DWI, ADC (axial view) without evidence of diffusion-restricting lesions. (C, D) FLAIR, SWI (axial view) demonstrating diffuse white matter signal hyperintensity without concomitant hemorrhage. (E, F) T1-MPRAGE before and after IV gadolinium (axial view) without evidence of gadolinium enhancement. DWI: diffusion-weighted imaging; ADC: apparent diffusion coefficient; FLAIR: fluid-attenuated inversion recovery; SWI: susceptibility-weighted imaging; MPRAGE: magnetization-prepared rapid gradient echo

He returned 10 days later following a witnessed seizure consisting of upper and lower body tonic-clonic movements at home. Per family members, who witnessed the event, the patient became unresponsive with upper and lower body jerking and a period of confusion afterward. He did not have any headaches, weakness, or confusion since his discharge and prior to this event. Repeat MRI brain demonstrated extensive non-enhancing white matter signal abnormalities and LP re-demonstrated lymphocytic pleocytosis with 14 nucleated cells and 99% lymphocytic predominance (Figure 3). Unfortunately, further markers for central nervous system (CNS) inflammation, such as oligoclonal banding, IgG index, and interleukin-6 (IL-6) were not sent. Infectious studies demonstrated positive West Nile Virus IgG in the CSF and the serum with negative IgM in the CSF and serum. The paraneoplastic panel returned negative. Given a history of frequent travel to and from Jamaica, and a previously unexplained transitory febrile episode four months prior to his initial presentation, his syndrome was consistent with sequelae from previous West Nile virus encephalitis, as in post-infectious encephalitis. 

**Figure 4:**
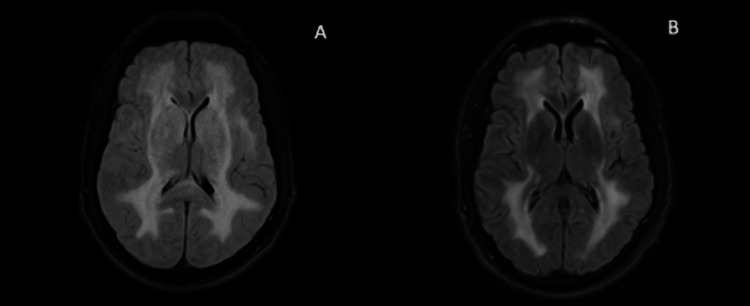
Comparison between MRI images (A) Initial MRI FLAIR with diffuse leukoencephalopathy. (B) Repeat MRI FLAIR nine months following initial presentation that demonstrates interval resolution of white matter signal change, especially in the basal ganglia and thalami. FLAIR: fluid-attenuated inversion recovery

He had a repeat MRI brain done three and nine months following his initial presentation, which revealed an interval decrease in abnormal signal change (Figure 4). He also had interval electroencephalogram (EEG) without seizures or epileptiform patterns. His headaches completely resolved, and he did not have any recurrent seizures on an anti-seizure regimen of levetiracetam at his nine-month follow-up visit.

## Discussion

The neurologic involvement of WNV infection is often the most significant effect. Symptoms are heterogeneous, varying from fatigue and mild weakness to cognitive, sensory, and focal motor deficits. About 35% of patients demonstrate complete recovery [10]. There is literature that suggests improved outcomes in patients with diffusion restriction changes on MRI without any other associated signal intensity abnormalities on FLAIR or T2WI [11,12]. Diffusion restriction has canonically been explained by neural cell death secondary to failure of adenosine triphosphate production. However, studies show that diffusion restriction need not imply cell death, especially when not associated with signal intensity abnormalities on other MR sequences. As such, WNV encephalitis cases with DWI changes, but not FLAIR changes, have been found to have better prognosis [9].

Our case is striking for the diffuse signal change present on FLAIR imaging, not associated with DWI signal change, and the positive clinical outcome. This stands in contrast to previous studies in which of the three patients with hyperintensities on FLAIR images and T2WIs, two died and one had severe residual neurologic deficits [9]. To our knowledge, there are no robust statistics for prognosis based on imaging that exist in the literature.

We did not note any WNV predilection for specific brain structures. Our patient’s MRI demonstrated a signal change in the frontal, parietal, and temporal lobes as well as the thalamus and brainstem, consistent with previous reports [9,12]. The imaging in our case is particularly striking given the diffuse FLAIR changes demonstrated throughout the white matter consistent with leukoencephalopathy. Initial radiographic differential diagnoses included toxic, metabolic, and hereditary etiologies. However, given the rapidity in the onset of symptoms, and the patient’s travel history to Jamaica with exposure to mosquitoes, WNV is an important infectious etiology to keep on the differential.

Ultimately, the diffuse pattern of signal change, coupled with positive IgG and negative IgM serologies, and the patient’s subacute and resolving clinical course make the diagnosis of sequelae from a previous WNV encephalitis most likely. Studies have shown that CNS invasion is not necessary for the development of neurologic sequelae, and in one case, 27% of patients diagnosed with WNF without being diagnosed with WNND had neurologic abnormalities three years later [13]. It remains unclear whether neurologic sequelae that occur after WNF are due to the peripheral inflammatory response that leads to neurologic malfunction or direct CNS invasion [14].

His imaging demonstrated improvement from presentation to subsequent imaging nine months later. These findings indicate ongoing and active inflammation at his initial presentation. WNV-specific IgM antibodies usually persist for 30 to 90 days, so it remains a possibility that our patient cleared IgM antibodies by the time of his presentation if the infection was four months prior. Unfortunately, reverse transcription-polymerase chain reaction (RT-PCR) for WNV was not sent, although sensitivity for this testing remains low. One limitation of our study is that antibodies induced by other flavivirus infections, such as dengue virus and St. Louis Encephalitis virus, which may show cross-reactivity with the WNV assay, were not tested.

It is unclear why our patient would develop neurologic sequelae from WNV infection, but evidence has linked old age [15] and concurrent chronic diseases, such as hypertension [16], as increasing his risk of sequelae. There is mixed evidence on whether the severity of the initial presentation is linked to chronic neurologic dysfunction. One study shows that increased severity, including hospitalization, does not increase risk [17]. Long-term outcomes can be varied [18].

The pathogenesis for post-infectious encephalitis remains to be elucidated. WNV is not known to demonstrate tropism for specific brain areas and has been found in many different brain regions, including the hippocampus, cerebellum, basal ganglia, thalamus, midbrain, pons, and cortex [15]. Histologic studies show that infection causes inflammatory cell infiltration with cell death, gliosis, reactive astrocytosis, and perivascular cuffing [19]. In one study on the long-term neuromuscular effects of WNV, neurophysiology studies showed both active and chronic denervation years after the initial infection, indicating an ongoing inflammatory process [20]. These findings are thought to be due to excitotoxic mechanisms or persistent viral infection. Another study that correlated long-term neuropsychological testing with MRIs of patients who survived WNV infection at least three years prior, showed cortical thinning in multiple brain regions including the posterior cingulate cortex, superior frontal cortex, and para-hippocampal region [21]. It is not known whether these central and peripheral nervous system sequelae are related to low-level viral replication or persistently elevated levels of pro-inflammatory cytokines. Expanded research is necessary to understand the underlying pathogenesis of WNV post-infectious encephalitis and its long-term neurologic sequelae.

## Conclusions

Our case demonstrates that despite striking imaging findings, it is difficult to assess the prognosis for WNV encephalitis, and there may be a clinico-radiological mismatch. Our patient continues to do well almost one year from the initial diagnosis despite the remarkable widespread leukoencephalopathy present on MRI. Patients may experience long-term sequelae of infection up to three years from the initial disease, and so should be monitored closely after the inciting event.

## References

[1] Sejvar JJ (2016). West Nile Virus infection. Microbiol Spectr.

[2] Chambers TJ, Halevy M, Nestorowicz A, Rice CM, Lustig S (1998). West Nile virus envelope proteins: nucleotide sequence analysis of strains differing in mouse neuroinvasiveness. J Gen Virol.

[3] Saiz JC, Martín-Acebes MA, Blázquez AB, Escribano-Romero E, Poderoso T, Jiménez de Oya N (2021). Pathogenicity and virulence of West Nile virus revisited eight decades after its first isolation. Virulence.

[4] Campbell GL, Marfin AA, Lanciotti RS, Gubler DJ (2002). West Nile virus. Lancet Infect Dis.

[5] Mostashari F, Bunning ML, Kitsutani PT (2001). Epidemic West Nile encephalitis, New York, 1999: results of a household-based seroepidemiological survey. Lancet.

[6] Granwehr BP, Lillibridge KM, Higgs S, Mason PW, Aronson JM, Campbell GA, Barrett AD (2004). West Nile virus: where are we now. Lancet Infect Dis.

[7] Nash D, Mostashari F, Fine A (2001). The outbreak of West Nile virus infection in the New York City area in 1999. N Engl J Med.

[8] Rosas H, Wippold FJ (2003). West Nile virus: case report with MR imaging findings. Am J Neuroradiol.

[9] Ali M, Safriel Y, Sohi J, Llave A, Weathers S (2005). West Nile virus infection: MR imaging findings in the nervous system. AJNR Am J Neuroradiol.

[10] Sampathkumar P (2003). West Nile virus: epidemiology, clinical presentation, diagnosis, and prevention. Mayo Clin Proc.

[11] Nouranifar RK, Ali M, Nath J (2003). The earliest manifestation of focal encephalitis on diffusion-weighted MRI. Clin Imaging.

[12] Kobata R, Tsukahara H, Nakai A (2002). Transient MR signal changes in the splenium of the corpus callosum in rotavirus encephalopathy: value of diffusion-weighted imaging. J Comput Assist Tomogr.

[13] Petropoulou KA, Gordon SM, Prayson RA, Ruggierri PM (2005). West Nile virus meningoencephalitis: MR imaging findings. AJNR Am J Neuroradiol.

[14] Weatherhead JE, Miller VE, Garcia MN, Hasbun R, Salazar L, Dimachkie MM, Murray KO (2015). Long-term neurological outcomes in West Nile virus-infected patients: an observational study. Am J Trop Med Hyg.

[15] Fulton CD, Beasley DW, Bente DA, Dineley KT (2020). Long-term, West Nile virus-induced neurological changes: a comparison of patients and rodent models. Brain Behav Immun Health.

[16] Lindsey NP, Staples JE, Lehman JA, Fischer M (2010). Surveillance for human West Nile virus disease - United States, 1999-2008. MMWR Surveill Summ.

[17] Lindsey NP, Staples JE, Lehman JA, Fischer M (2012). Medical risk factors for severe West Nile Virus disease, United States, 2008-2010. Am J Trop Med Hyg.

[18] Carson PJ, Konewko P, Wold KS, Mariani P, Goli S, Bergloff P, Crosby RD (2006). Long-term clinical and neuropsychological outcomes of West Nile virus infection. Clin Infect Dis.

[19] Armah HB, Wang G, Omalu BI (2007). Systemic distribution of West Nile virus infection: postmortem immunohistochemical study of six cases. Brain Pathol.

[20] Athar P, Hasbun R, Nolan MS, Salazar L, Woods SP, Sheikh K, Murray KO (2018). Long-term neuromuscular outcomes of West Nile virus infection: a clinical and electromyographic evaluation of patients with a history of infection. Muscle Nerve.

[21] Murray KO, Nolan MS, Ronca SE (2018). The neurocognitive and MRI outcomes of West Nile virus infection: preliminary analysis using an external control group. Front Neurol.

